# Probiotics beyond the farm: Benefits, costs, and considerations of using antibiotic alternatives in livestock

**DOI:** 10.3389/frabi.2022.1003912

**Published:** 2022-10-12

**Authors:** Kyle R. Leistikow, Rachelle E. Beattie, Krassimira R. Hristova

**Affiliations:** ^1^ Department of Biological Sciences, Marquette University, Milwaukee, WI, United States; ^2^ U.S. Geological Survey, Columbia Environmental Research Center, Columbia, MO, United States

**Keywords:** livestock, probiotic, antibiotic resistance, microbiome, regulation, *Bacillus*, environment

## Abstract

The increasing global expansion of antimicrobial resistant infections warrants the development of effective antibiotic alternative therapies, particularly for use in livestock production, an agricultural sector that is perceived to disproportionately contribute to the antimicrobial resistance (AMR) crisis by consuming nearly two-thirds of the global antibiotic supply. Probiotics and probiotic derived compounds are promising alternative therapies, and their successful use in disease prevention, treatment, and animal performance commands attention. However, insufficient or outdated probiotic screening techniques may unintentionally contribute to this crisis, and few longitudinal studies have been conducted to determine what role probiotics play in AMR dissemination in animal hosts and the surrounding environment. In this review, we briefly summarize the current literature regarding the efficacy, feasibility, and limitations of probiotics, including an evaluation of their impact on the animal microbiome and resistome and their potential to influence AMR in the environment. Probiotic application for livestock is often touted as an ideal alternative therapy that might reduce the need for antibiotic use in agriculture and the negative downstream impacts. However, as detailed in this review, limited research has been conducted linking probiotic usage with reductions in AMR in agricultural or natural environments. Additionally, we discuss the methods, including limitations, of current probiotic screening techniques across the globe, highlighting approaches aimed at reducing antibiotic usage and ensuring safe and effective probiotic mediated health outcomes. Based on this information, we propose economic and logistical considerations for bringing probiotic therapies to market including regulatory roadblocks, future innovations, and the significant gaps in knowledge requiring additional research to ensure probiotics are suitable long-term options for livestock producers as an antibiotic alternative therapy.

## Introduction

**Figure 1:**
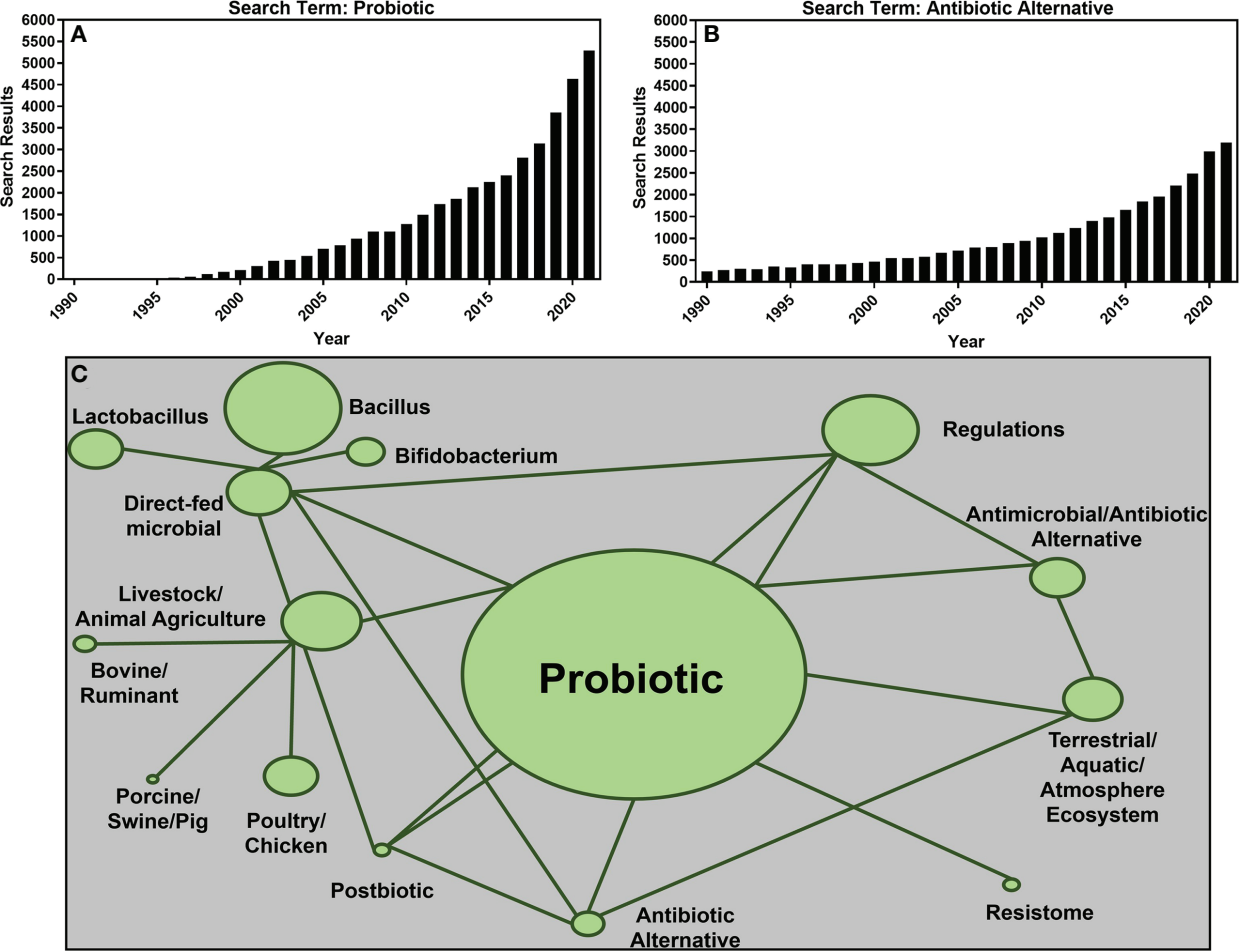
The number of publication search results returned from the National Center for Biotechnology Information (NCBI) PubMed repository using key terms “probiotic” **(A)** and “antibiotic alternative **(B)** between years 1990 and 2021. A network of the most common search terms used for this review is shown in **(C)**. The size of the network nodes is representative of the number of papers containing the term with larger nodes representing more papers included in this review. Linkages between nodes represent search terms combined to source papers for this review.

In 2015, the World Health Organization declared antimicrobial resistance (AMR) a serious global public health threat (World Health Organization, 2015). Researchers worldwide were encouraged to focus on improving awareness, understanding, and surveillance of AMR in addition to developing mitigation measures to reduce the use of antibiotic drugs for human and animal health (World Health Organization, 2015). This reduction in antibiotics use was, and is, intended to reduce the dissemination of antibiotics into the environment. However, to reduce antibiotic use, a transition to current antibiotic alternative therapies or development of new alternative antibiotic therapies is needed to combat the diseases antibiotics currently treat (Baker et al., 2018). One such alternative therapy is probiotics. Defined by the World Health Organization as, “live microorganisms which when administered in adequate amounts confer a health benefit to the host” (FAO/WHO, 2001), probiotics have routinely been suggested as antibiotic alternatives for a variety of human and animal disease states and conditions (Santacroce et al., 2019; Alayande et al., 2020; Al-Shawi et al., 2020; Green et al., 2020). Research investigating antibiotic alternatives such as probiotics has grown exponentially since the early 2000s with a basic National Center for Biotechnology Information (NCBI)PubMed search of the term “probiotics” returning just 212 results in 2000 compared to 2,254 results in 2015 and 5,286 results in 2021 alone (**Figure 1A**) and the term “antibiotic alternative” seeing a similar increase in interest with 463 results in the year 2000, 1,656 results in 2015, and 3,196 results in 2022 (**Figure 1B**).

One important consideration for antibiotic alternative therapies including probiotics is the degree to which their use reduces antibiotic resistance. However, this outcome is rarely studied; instead, most research in this area focuses on the ability of the antibiotic alternative to treat and prevent disease (Defoirdt et al., 2011; Allen et al., 2013; Nair D et al., 2018). Thus, the goal of this review is to provide a better understanding of the benefits and consequences of using probiotics as an antibiotic alternative therapy on AMR in the environment. Because probiotics are used broadly across human and animal medicine, we focus this review specifically on the use of probiotics for livestock to prevent and treat a variety of disease states or improve animal performance. We pay special attention to less understood outcomes of probiotic use including the pros and cons of using probiotics on the animal gut microbiome, the impact of probiotic use in animal agriculture on the environment, regulations governing the use of probiotics worldwide, and the financial implications of using probiotics versus antibiotic drugs in animal agriculture. Together, this review summarizes critical research regarding the feasibility of using probiotics as an antibiotic alternative and provides suggestions for future research needed to fill knowledge gaps surrounding probiotic use and environmental AMR.

Research articles included in this review were sourced using the search terms or a combination thereof found in **Figure 1C** using both Google Scholar and NCBI’s PubMed database. To be included, articles must have been published primarily within the previous five years (2017–2022) with the exception of reviews or regulatory guidance documents. Using the outlined search terms and criteria, a total of 204 research papers and governmental reports or publications were selected (**Figure 1C**).

## Probiotics as an antibiotic alternative for animal agriculture

The discovery and subsequent industrial manufacturing of antibiotic compounds revolutionized healthcare in the 20^th^ century. However, despite their efficacy, overuse and misuse of antibiotics has led to an increase in bacterial AMR, a serious public health threat. According to the World Health Organization, AMR accounted for an estimated 700,000 deaths worldwide in 2019, a number that is expected to surpass 10 million by 2050 if immediate measures are not taken. In the United States alone, 2.5 million AMR infections resulted in an estimated annual economic cost of more than $55 billion (Ahmad and Khan, 2019; Centers for Disease Control and Prevention (U.S.), 2019).

In modern day livestock production, sub-therapeutic doses of antibiotics have been applied to animal feeds to both prevent diseases and improve growth performance; however, bacteria have evolved a variety of unique strategies to develop resistance to these compounds, and antibiotic-resistant bacteria (ARB) emerged in the animal microbiome. The bilateral movement of ARB occurs regularly between livestock operations and human communities through air, water, direct physical contact, and, perhaps most notably, the food chain (Stanton, 2013). This transmission poses a significant risk to human health and has been demonstrated in a variety of ways including antibiotic residues making their way into meat, unapparent carriage of ARB out of animal facilities, and plasmid exchange from ARB in the microbiota of food grade animals to common human pathogens (Ramatla et al., 2017; Rousham et al., 2018). These concerns have encouraged new efforts to investigate antibiotic alternative therapies (Cheng et al., 2014; Sang and Blecha, 2015). Antibiotic alternatives are broadly defined as any substance that can prevent or reduce the need for antimicrobial drugs – these can include vaccines, phytochemicals, organic acids, phage, and other non-disease-causing bacteria (Kumar et al., 2021).

The first recorded use of live microorganisms in food dates back to 2000 BCE when humans discovered how to preserve milk and transform it into fermented dairy products using unidentified bacteria and yeast (Ozen and Dinleyici, 2015). The intentional use of live microorganisms (now termed probiotics) in livestock production began in the 1970s and has been increasingly studied in a variety of both veterinary and human health applications (Goldenberg et al., 2015; Buntyn et al., 2016; Markowiak and Śliżewska, 2018; Rainard and Foucras, 2018; Vieco-Saiz et al., 2019). In small ruminants, cattle, poultry, and swine, probiotics improve animal health, immunity, and growth efficiency (Abd El-Tawab et al., 2016; Alayande et al., 2020). The mechanisms by which these outcomes are obtained are largely strain dependent but can include the stabilization of disturbed intestinal microbial communities, bacteriocin production and competitive exclusion of pathogens, improving intestinal epithelium integrity and permeability, modulation of fecal enzymatic activities, production of short-chain and branched-chain fatty acids, modulation of the immune system, and interaction with the gut-brain axis through the regulation of endocrine and neurologic functions (Plaza-Diaz et al., 2019). In this section, we detail the mechanism of action of many common probiotics used for livestock as well as the impact on the animal microbiome and resistome.

## Defining effective probiotics for animal agriculture

Probiotics have been shown to simultaneously prevent and treat a variety of diseases by controlling pathogens directly through competitive interactions or indirectly by stimulating the host immune response (Hossain et al., 2017; Raheem et al., 2021). Probiotics have also been shown to improve animal performance; however, efficacy tends to be variable across individual livestock operations and is often affected by external factors such as weather or feed composition (Talkington et al., 2017). The reproducibility required of probiotics is further complicated by multiple potential mechanisms of action (i.e., the molecular processes that generate the desired effect) that are often not thoroughly understood at the time of administration. At a minimum, probiotics should provide both prophylactic and therapeutic efficacy if they are going to assist in controlling ARB and influence disease outcomes. Genetic information and bioinformatic analyses could help ensure each probiotic strain is safe and appropriately vetted for mobile genetic elements harboring antibiotic resistance genes (ARGs), virulence factors, and other pertinent traits related to the strain’s functioning *in vivo*. It is estimated that by 2050, the global demand for food will increase by 100-110% (Tilman et al., 2011), and the livestock industry will rely heavily on probiotics to not only combat antibiotic resistance, but also to improve production efficiencies (Gilchrist et al., 2007).

Bacterial genera commonly utilized as probiotics include, but are not limited to, *Lactobacillus, Bifidobacterium, Enterococcus, Streptococcus, Pediococcus*, and *Bacillus (* Fijan, 2014). While lactic acid producing probiotics, namely those belonging to *Bifidobacterium, Enterococcus, Streptococcus, Pediococcus*, and *Lactobacillus genera*, have long been used in animal husbandry (Deng et al., 2021), *Bacillus* species are gaining interest as probiotics for their ability to produce an array of antimicrobials and immune modulating signaling peptides and improved shelf life stability owing to the spore forming ability of *Bacillus*. Probiotic *Bacillus* species have been shown to modulate the gut microbial composition of poultry and livestock to reduce indicators of disease (Kim et al., 2019; Hernandez-Patlan et al., 2019), improve nutrient digestibility and growth performance (Park et al., 2020; Lewton et al., 2021; Tian et al., 2021), and reduce the need for antibiotics (Luise et al., 2022). A thorough review by Mingmongkolchai and Panbangred outlines the *Bacillus* species currently used in the poultry and livestock sectors (Mingmongkolchai and Panbangred, 2018).

## Impact of probiotic use on the animal microbiota

The intestinal microbiota of food producing animals fluctuates throughout development (Wang et al., 2019; Ngunjiri et al., 2019), and early bacterial colonization of the gastrointestinal (GI) tract of swine and poultry has been shown to play an important role in shaping microbial composition and future host performance (Mach et al., 2015; Mancabelli et al., 2016). Microbial composition significantly influences host health, immunity, nutrient digestion, and feeding requirements (Clavijo and Flórez, 2018; Kumar et al., 2019). Numerous studies have demonstrated that the establishment of commensal and mutualistic probiotic microorganisms may inhibit disease-causing bacteria found in the same host microbial environment (Varankovich et al., 2015). Metagenomic analyses have also revealed probiotic supplementation increased the microbial diversity and richness in the swine GI tract both in the presence and absence of an enteric challenge in a variety of stages of development (Zhang et al., 2017). When compared to an antibiotic growth promoter, Wang and colleagues showed supplementation with probiotic species *Lactobacillus plantarum* PFM 105 increased production of short chain fatty acids while antibiotics did not provide this benefit (Wang et al., 2019). Shin et al. discovered that microbial diversity, richness, and the relative abundance of Firmicutes were higher in weaned piglets fed *L. plantarum* JDFM LP11 (Shin et al., 2019). Reviews by Azad (Azad et al., 2018) and Valeriano (Valeriano et al., 2017) outline a variety of opportunities *Lactobacillus* provides in the swine gut and discuss the role of these bacteria in pig performance, husbandry, and disease prevention.

In addition to *Lactobacillus* species, members of the *Bacillus subtilis* group are some of the most commercially important probiotics, used to produce vitamins, amino acids, antibiotics, and industrial enzymes (Harwood et al., 2018). They also produce a range of secondary metabolites, including polyketides, terpenes and siderophores, as well as ribosomal and non-ribosomally synthesized peptides (Caulier et al., 2019). These metabolites can damage the cell wall, cell membrane, impede intracellular processes, and disrupt communication networks in competing microorganisms (Tran et al., 2022), providing *B. subtilis* a competitive advantage in complex communities seen in the GI tract of livestock. *Bacillus* species have also been shown to beneficially modify the animal microbiota. Pigs fed *B. subtilis* DSM 32540 showed decreased coliform abundance in the mesenteric lymph nodes and reduced relative ileal abundance of multiple bacterial families known to contribute to enteric swine diseases (He et al., 2020). Broiler chickens fed *Bacillus amyloliquefaciens* showed increased microbial alpha diversity in the jejunum, ileum, and cecum resulting in improved immune responses and epithelial barrier integrity (Wang et al., 2021). However, these positive effects are not universally observed across livestock operations. Using an identical strain, probiotic effectiveness can be mitigated or enhanced if applied at different doses or in the presence of high or low metabolizable energy diet formulations (Krueger et al., 2020). These variables may help to explain conflicting performance outcomes across studies investigating identical probiotic strains.

**Figure 2:**
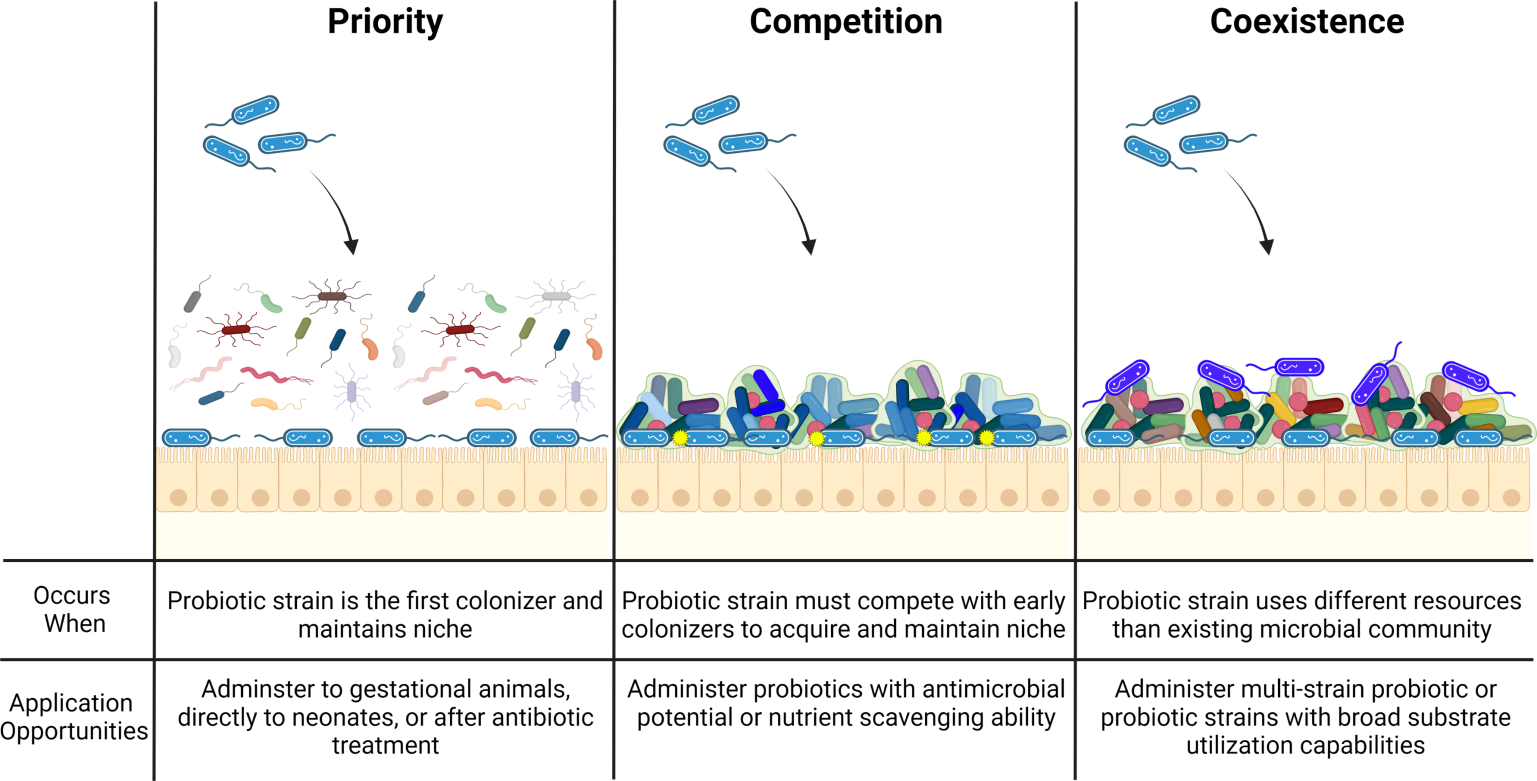
Ecological principles driving effective probiotic strain colonization. To effectively colonize the host gastrointestinal tract, probiotic strains (blue bacilli) must be administered to a naïve or depleted microbiome community (priority), produce antimicrobial products (yellow circles) to outcompete resident microbial strains (competition), or possess an ability to inhabit a broad ecological range (coexistence) so as to not compete with similar resident microorganisms (royal blue bacilli).

Researchers continue to investigate the effect of probiotic species on acute GI diseases; however, few longitudinal studies have been conducted to determine whether the treatment of such diseases using probiotics disrupts the microbiota in a way that puts the animal at risk for future diseases (Schoster et al., 2016; Zeineldin et al., 2019). Probiotic species such as *Lactobacillus plantarum, Enterococcus faecium*, and *Bacillus subtilis* are known to produce antimicrobial compounds with broad efficacy; however, there has been little investigation into the interaction of these probiotic derived compounds with the native gut microbiota. It is possible that unintended consequences such as competitive exchanges altering gut homeostasis may occur following the application of probiotic therapies. Therefore, bacterial competition also needs to be considered when selecting probiotic strains as long-term colonization (engraftment) is often a desirable outcome of probiotic supplements (Arshad et al., 2021) (**Figure 2**). Competitive outcomes in gut ecosystems depend on niche differences and are historically contingent on the order in which probiotic strains are introduced (Segura Munoz et al., 2022). This theory might explain why probiotics administered to gestational animals yield positive long term health outcomes for the resulting progeny (Baker et al., 2013; Veljović et al., 2017). Recent evidence suggests that engraftment may depend on the pre-treatment microbiota composition, especially the absence of closely related species. The resident microbiota influences engraftment of incoming species, likely through competitive exclusion where newly-arriving species cannot coexist with established species if they occupy exactly the same niche (and are competing for identical resources) (Maldonado-Gómez et al., 2016; Martínez et al., 2018). These phenomena have also given validity to the use of multi-strain probiotics (Lambo et al., 2021).

Though the mechanistic benefits associated with probiotics are vast, the reproducibility of these interventions is largely dependent on host physiology, diet and feeding frequency, variation in sampling methodologies, and a myriad of other environmental factors (Johnson et al., 2018; Fomenky et al., 2018). Therefore, microbially derived small molecules, termed postbiotics, are providing an alternative approach to probiotics by both eliminating the perceived concern of administering live bacteria and ensuring greater reproducibility by using a more defined quantity of active compounds. Postbiotic compounds have been shown to improve gut physiological processes and even improve adaptive immunity (Villena et al., 2016; Villena et al., 2018; Iida et al., 2019). Postbiotic compounds have been shown to improve gut physiological processes and even improve adaptive immunity. In addition to low toxicity concerns and improved shelf life stability, many postbiotics have defined chemical structures and mechanisms that make them highly therapeutically attractive. However, the cost and expertise required to determine metabolite structure and function is a hinderance for many researchers interested in this technology; therefore, despite an emerging interest in this application strategy, little research has been conducted to understand the impact of postbiotics on the animal microbiota. For a comprehensive review on postbiotics, their beneficial effects on the host, and their interaction with host cells please refer to Zamojska (Zamojska et al., 2021), Nataraj (Nataraj et al., 2020), and Teame et al (Teame et al., 2020).

## Impact of probiotic use on the animal microbiome and resistome

Extensive work has been conducted to identify how probiotics employ mechanisms to regulate both commensal and pathogenic bacterial populations, but insufficient attention has been given to the impact probiotics have on the development of resistance in the larger bacterial populations they intend to target (Willing et al., 2018). Similar to other microorganisms, probiotic strains are not exempt from intrinsic resistance or acquiring ARGs - a method used by all bacteria to survive and increase their ecological fitness (van Schaik, 2015). Given their shared microbial environment in the GI tract, the risk of pathogenic microorganisms acquiring ARGs from probiotic species and vice versa exists (Imperial and Ibana, 2016; Li et al., 2020); therefore, probiotic efficacy may be undermined by tolerance or resistance development in these larger populations. Several physiological and biochemical mechanisms have been established as drivers of this developing resistance (Aslam et al., 2018). A summary of intrinsic and acquired resistance in four genera frequently used as probiotics is found in **Table 1**.

**Table 1:** Summary of intrinsic and acquired resistance in four major probiotic genera used for disease treatment and prevention in livestock (Gueimonde et al., 2013; Li et al., 2020; Ferchichi et al., 2021).

Probiotic Genus	Intrinsic Resistance	Acquired Resistance	Primary Transfer Method of Resistance Determinants
*Bacillus*	Aminoglycosides, Chloramphenicol	Macrolides, Tetracyclines	Plasmid
*Lactobacillus*	Vancomycin, Kanamycin, Gentamicin, Streptomycin, Metronidazole, Nalidixic Acid	Chloramphenicol, Macrolides, Tetracyclines	Plasmid, Transposon, Mobile Genetic Element
*Bifidobacterium*	Mupirocin, Aminoglycosides, Vancomycin, Kanamycin, Gentamicin, Streptomycin, Metronidazole, Norfloxacin, Polymyxin B, Nalidixic Acid	Macrolides	Transposon
*Enterococcus*	Vancomycin, Streptomycin, Cephalosporins, Aminoglycosides, Erythromycin	Ampicillin, Chloramphenicol, Erythromycin, Fluoroquinolones, Penicillin, Tetracyclines, Aminoglycosides	Plasmid, Transposon, Mobile Genetic Element

Multiple probiotic genera possess AMR phenotypes (Selvin et al., 2020). Tetracycline resistance has been extensively studied in *Bifidobacterium*, and genes synonymous with tetracycline resistance have been detected in several species (Aires et al., 2007; Ammor et al., 2008; Aires et al., 2009). Certain *Lactobacillus* species commonly employed as probiotics have exhibited intrinsic resistance to vancomycin, an antibiotic used to treat clinical MRSA infections (Mater et al., 2008). Perhaps more concerning are the findings of intra and inter genus transmission of various antibiotic resistant determinants. Previous work with *Lactobacillus* species have demonstrated that this microorganism can both transmit to and receive ARGs from known human pathogens (Tannock et al., 1994; Drago et al., 2011; Klein, 2011). In fact, a genus-wide assessment of ARGs in *Lactobacillus* species revealed the majority of genomic ARGs were flanked by mobile genetic elements with potential for horizontal gene transfer (HGT) (Campedelli et al., 2019). These findings provide evidence for regulatory authorities to re-evaluate and potentially revise the safety assessment guidelines for Lactobacilli entering the food chain as probiotics.

## Do probiotics contribute to or reduce antimicrobial resistance in livestock?

Although the presence of ARGs and HGT capabilities in probiotic species is concerning, additional data suggest the carriage and transmissibility of ARGs discovered in probiotic species is reduced based on gene location. For example, *Bifidobacterium* are intrinsically resistant to mupirocin and high concentrations of aminoglycosides (Gueimonde et al., 2013). However, recent work by Duranti and colleagues (Duranti et al., 2017) demonstrated that resistant probiotic strains of *Bifidobacterium* did not always contain predicted gene mobility, indicating these strains may not be able to confer these intrinsic resistances to other bacterial species. Similar to Duranti’s findings, Sato and Iino determined that streptomycin and erythromycin resistant *Bifidobacterium* strains were unable to transfer this resistance to neighboring bacteria through traditional mobile genetic elements (Sato and Iino, 2010). Bozdogan and colleagues (Bozdogan et al., 2003) identified that the presence of the aadD2 gene in *Bacillus clausii* resulted in kanamycin, tobramycin, and amikacin resistance, but this chromosomally located sequence was not transferable by conjugation experiments. These findings have also been investigated *in vivo*, where human participants showed no lingering tetracycline resistance in stool samples after being administered *Lactobacillus reuteri* carrying a tetW plasmid (Egervärn et al., 2010). This result may be attributed to population diversity brought about by the probiotic, since this selection for antimicrobial resistance is reduced when embedded in a natural microbial community (Klümper et al., 2019).

Interestingly*, B. subtilis* possesses an ability to identify and discriminate between closely related strains prior to initiating gene transfer events (Stefanic et al., 2021) and targeted antimicrobial production (Maan et al., 2022), suggesting social interactions within this species can override mechanistic barriers to horizontal gene transfer. Evidence also exists demonstrating the administration of *B. subtilis* may help reduce ARG presence by inhibiting pathogens, namely F18 enterotoxigenic *Escherichia coli*, known to carry and transmit a variety of mobile genetic elements harboring ARGs (Kim et al., 2019). It is worth noting that sporulating microorganisms like *Bacillus* are among the most prolific producers of antimicrobial compounds, in part reflecting aspects of the sporulation process itself which requires a significant portion of the population be sacrificed to provide the nutrients required of sporulating cells. Therefore, the competitive exclusion principle afforded by probiotics can also serve as a mechanism to reduce AMR selection. Using pig fecal microbial communities, Klümper and colleagues revealed that certain communities imposed a fitness cost of maintaining gentamicin and kanamycin resistant *E. coli* even in the presence of increasing antibiotic concentrations (Klümper et al., 2019).

Researchers and medical professionals continue to ask whether antibiotic/probiotic combination therapies are warranted (Kerna, 2018; Li et al., 2018; Wealleans et al., 2018), but conducting this type of intervention requires probiotic strains exhibit some level of resistance to the antibiotic they are paired with. This strategy results in screening efforts designed specifically to select for probiotics with AMR phenotypes (Galopin et al., 2009; Hammad and Shimamoto, 2010). Furthermore, animals treated with these resistant probiotics risk becoming a possible source of ARGs for human consumers after ingestion of meat or dairy products (Forslund et al., 2013; Hu et al., 2014; Huddleston, 2014; Woolhouse et al., 2015; Montassier et al., 2021). Despite a multitude of animal models revealing improved immunological outcomes after probiotic administration, the impact of probiotics applied to livestock on the consumer immune profile has yet to be fully investigated (Patel et al., 2015). Human consumption of probiotics has yielded a variety of positive clinical outcomes; however, much less vetting is performed on probiotics intended for livestock than those intended for human use. Additionally, animals that consume probiotics inevitably excrete these microorganisms into manure lagoons or pits. These pits are often a primary source of fertilizer for neighboring crop production systems and the contamination of such fertilizer with ARGs frequently results in dissemination to recreational waterways of surrounding local communities (Beattie et al., 2018). As such, there is a need to review existing studies and to perform new research to ensure the safety of probiotics and evaluate their impact on ARG prevalence and dissemination in animal, human, and downstream environments.

## Impact of probiotic use on ecosystems and environmental antimicrobial resistance

In recent years, substantial amounts of research have focused on the impacts of antibiotics used for animal agriculture on the downstream environment. Multiple research and review articles clearly highlight the negative repercussions of excessive antibiotic use across various environments and the relative ease with which these drugs disseminate from their point of origin (Berglund, 2015; Saima et al., 2020; Larsson and Flach, 2022). A major goal of transitioning to probiotics in animal agriculture is to reduce the broader environmental consequences of antibiotic use. Thus, a thorough understanding of the dissemination and impact of animal fed probiotics and their capability to reduce environmental AMR is necessary.

## Terrestrial ecosystems

Soil microbiota are critical for terrestrial ecosystem health. However, degradation of the soil microbiota is becoming more common worldwide due in part to unsustainable agricultural practices. To combat the loss of this critical resource, plant growth promoting bacteria (i.e., soil probiotics) are being incorporated into soils as a sustainable crop agriculture practice. Probiotics targeted for soil health and/or plant growth include multiple strains of *Bacillus (* Akinrinlola et al., 2018; Hashem et al., 2019) and *Pseudomonas (* Anderson and Kim, 2018) bacteria in addition to strains with the potential to minimize the activity of pathogenic microorganisms such as *Bacillus* and *Lactobacillus* spp (Tsuda et al., 2016; de Souza Vandenberghe et al., 2017; Kiesewalter et al., 2021). These probiotic bacteria are particularly beneficial in agricultural soils that have been degraded by common practices including chemical fertilization, tilling, and exposed soil surfaces (Jie et al., 2002; Glick et al., 2012). When applied to agricultural soils or crop cultivars, probiotic bacteria enhance plant growth through multiple mechanisms including nutrient acquisition and reduction of pathogens (Glick et al., 2012).

Common animal probiotic taxa including *Bacillus* spp. and *Lactobacillus* spp. are also beneficial in terrestrial ecosystems (Quattrini et al., 2018; Sansinenea and Bacillus, 2019; Tiwari et al., 2019; Fan et al., 2021). If a portion of these probiotic species applied to livestock survive the animal intestinal tract and are excreted, it stands to reason that manure from these animals could then be applied to area cropland for an additional probiotic benefit. However, to the best of our knowledge, limited data are available regarding the excretion rate or concentration of direct fed probiotics in animal feces. Additionally, even if probiotic species known to benefit livestock, soils, and plants are supplied, positive effects across all groups may not occur as probiotic benefits are highly strain specific (McFarland et al., 2018). Lastly, the ability of many probiotic bacterial species to acquire and disseminate ARGs suggests that supplying inadequately screened probiotics to terrestrial environments may exacerbate the AMR issue further rather than result in a reduction of resistance in the environment.

## Aquatic ecosystems

Aquatic ecosystems support a wide variety of habitats that may be altered by anthropogenic contamination contributing to the decline of water quality worldwide. Water quality is a significant concern, especially in areas of intensive livestock farming (Burkholder et al., 2007; Li et al., 2015; Jacobs et al., 2019; Beattie et al., 2020). Runoff from agricultural fields has been shown to contain elevated levels of livestock contaminants including ammonia, nitrogen, and ARB (Sobsey et al., 2006; Beattie et al., 2018; Le et al., 2018; Tang et al., 2020). In addition to terrestrial livestock farming, aquaculture practices have been implicated in increased AMR (Reverter et al., 2020). Pressure on aquatic ecosystems from aquaculture practices is predicted to increase significantly in the next 10 years as increasing demand for food supplies necessitates a shift to fish and seafood (Anderson et al., 2019; Chan et al., 2019). To mitigate the consequences of expanding aquaculture, the application of probiotics has been suggested to improve both water quality and the growth and health of aquatic animals used for food (Ibrahem, 2015).

*Bacillus* spp. and *Lactobacillus* spp. have been widely used as probiotics in aquaculture for the removal of nitrogenous wastes and biogeochemical cycling (Chauhan and Singh, 2019). Specific species of these groups, such as *B. subtilis*, have been shown to increase support of both water quality and digestion in fish and shrimp aquaculture (Olmos et al., 2020). Other *Bacillus* spp. have been shown to reduce stress, prevent disease, and enhance growth in aquaculture systems (Kuebutornye et al., 2019). It stands to reason that the application of common groups of probiotics instead of antimicrobials for animal agriculture and aquaculture may help mediate the spread of antimicrobial resistance in the environment and may even help improve water quality locally. However, limited downstream research has been conducted to date to corroborate this assumed positive benefit.

## Atmosphere ecosystems

Atmospheric ecosystems are a relatively new consideration in the context of livestock pollution and AMR. However, recent research has shown that ARB and ARGs can be transmitted on airborne particles in areas near concentrated livestock farms including those practicing methods aimed at reducing AMR such as composting (Gao et al., 2018; Ma et al., 2019). These airborne particles can disseminate ARB far beyond the point of origin, potentially impacting health across the biosphere (de Rooij et al., 2019). Reducing the application and necessity of antibiotics in animal agriculture through the use of alternative therapies such as probiotics may result in a reduction in ARB and ARGs in the atmosphere; however, this area of research remains in early stages and additional work is needed to draw conclusions.

## Probiotic impact on environmental antimicrobial resistance

**Figure 3:**
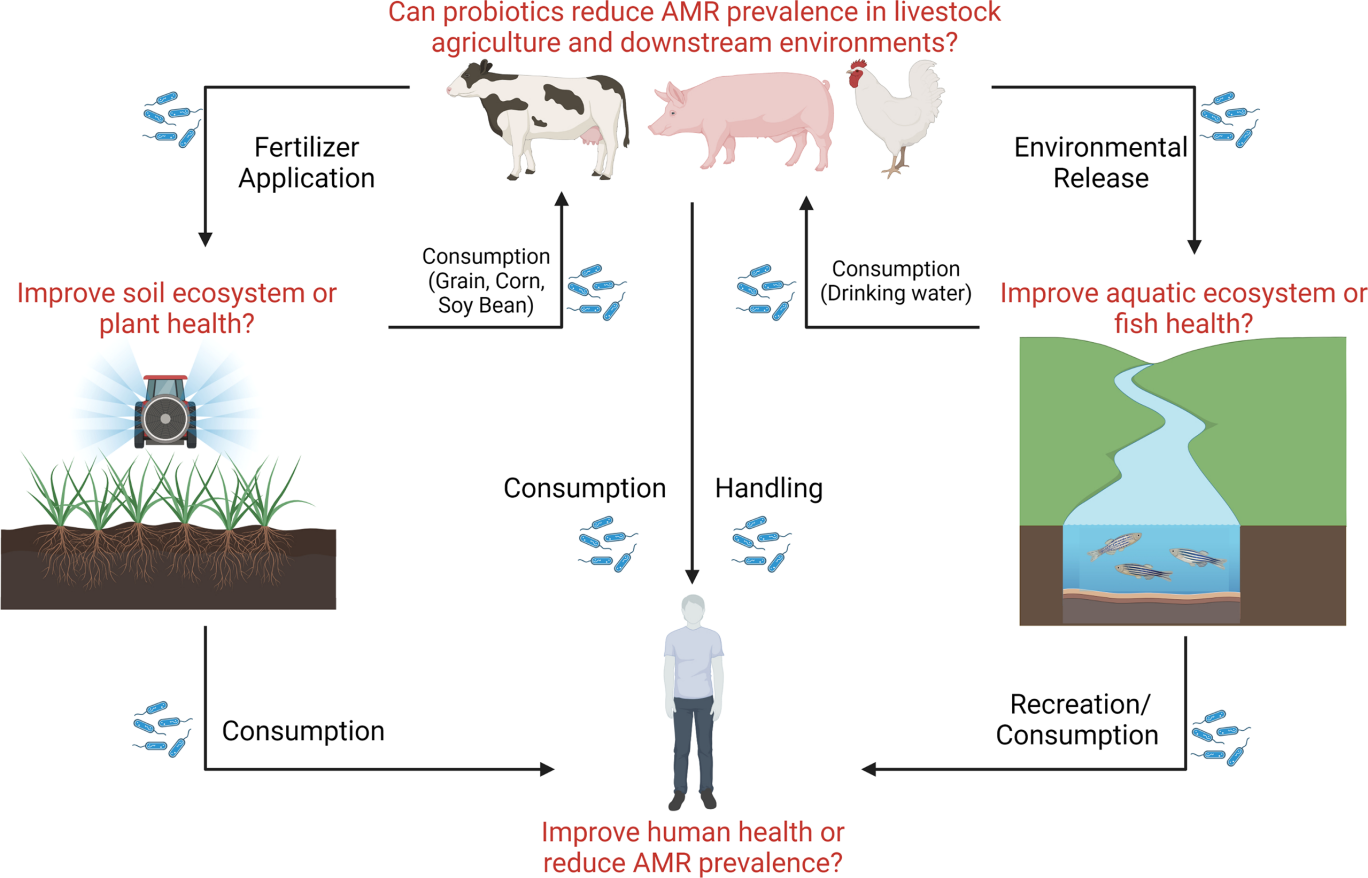
The potential impact of probiotic dissemination on ecosystem health. Probiotic research has attempted to assess strain efficacy in livestock, soil, plant, aquatic, and clinical environments, however few efforts have attempted to assess the impact of probiotics administered to livestock on downstream ecosystem health and AMR dissemination. Here, we show critical knowledge gaps that remain outstanding surrounding probiotic use (represented in the figure by blue bacilli) and the dissemination of environmental AMR.

One significant benefit of transitioning to antibiotic alternative therapies such as probiotics is a reduction of antibiotic usage in the clinic and veterinary medicine and thereby an assumed overall reduction in AMR in areas including in the environment. However, despite the frequent mention of this benefit in reviews and research articles on the topic, critical gaps in knowledge remain (**Figure 3**). Does the use of probiotics instead of antibiotics reduce the spread of AMR? The answer to this vital question remains unclear. In human medicine, probiotic cleaning protocols have been implemented to replace antimicrobial and chemical protocols in hospitals in Italy resulting in successful reduction in AMR (Caselli et al., 2019; D’Accolti et al., 2019). However, to the best of our knowledge, this is the only study clearly linking probiotic use to a reduction in AMR across human and animal medicine and the environment. Because probiotic species and strains have been shown to either carry ARGs or have the ability to acquire resistance, significantly more research in this area is necessary to ensure that the transition to probiotic use from antibiotics actually results in a reduction in AMR.

## Worldwide regulation of probiotics used for animal agriculture

The number of sequenced bacterial genomes has increased exponentially over the last two decades (Land et al., 2015), revealing extraordinary strain level genetic differences in probiotic species and providing valuable data needed for comprehensive probiotic evaluation prior to use. Despite a multitude of animal models revealing improved immunological outcomes after probiotic administration, the regulations regarding safety, efficacy, and sourcing differ substantially worldwide, causing consumer confusion and potentially misleading or even harmful outcomes. Additionally, less vetting is performed on probiotics intended for livestock than those intended for human consumption which may lead to the sale of probiotic strains containing transmissible ARGs or other mobile genetic elements, increasing the risk of environmental dissemination of ARGs and ARB. Here, we detail the similarities and differences in the regulation of probiotics for livestock in five regions across the globe with the highest reported antibiotic usage for livestock and/or livestock exports (Van Boeckel et al., 2015; European Medicines Agency, 2017; Van Boeckel Thomas et al., 2017), focusing on factors which may improve or contribute to AMR in the environment. Additionally, we note which regions utilize the “precautionary principle” requiring proof of safety prior to use and those that assume safety of new products until otherwise proven unsafe. We end this section with a “proof of concept” demonstrating the complexities of probiotic sourcing and usage in the United States compared to other regions using *Bacillus subtilis* as an example (**Table 2**).

**Table 2:** Regulations governing probiotic use in animal feed in five regions across the globe.

Region	Agricultural Feed Governing Body	Probiotic Governing Body (if applicable)	Criteria Needed for Approval	Taxonomic Level for Approval	Is strain safety data required?	Is product safety data required?	Would *Bacillus subtilis* be approved for use in animal feed?
United States of America	U.S. FDA	U.S. FDA, AAFCO	safe history of use; assumption of safety principle	species	No	Yes	Yes
Canada	CFIA	CFIA	strain identification, pathogenicity testing, antimicrobial production, antimicrobial resistance, safety, and efficacy with one controlled trial	strain	Yes	Yes	Not at the species level
European Union	EFSA	EFSA	whole genome study, functional capability including antimicrobial production and resistance, virulence, etc., three long and/or short-term controlled safety and efficacy studies	strain	Yes	Yes	Not at the species level
Brazil	MAPA	ANVISA (human food probiotics)	N/A: In development for agricultural products	N/A	N/A	N/A	N/A
China	MARA	MARA	safety statement from manufacturer/manufacturing facility	species or strain depending on country of origin	No	No	Yes, with safety statement from United States or European Union

U.S. FDA, United States Food and Drug Administration; AAFCO, Association of American Feed Control Officials; CFIA, Canadian Food Inspection Agency; EFSA, European Food Safety Authority; MAPA, Ministry of Agriculture, Livestock and Food Supply; ANVISA, Brazilian Health Regulatory Agency; MARA, Ministry of Agriculture and Rural Affairs.

## United States

Probiotics used for livestock, also termed direct-fed microbials in the United States, are regulated as feed ingredients at the federal level by both the Center for Veterinary Medicine (a subsidiary of the U.S. Food and Drug Administration) and the American Association of Feed Control Officials (AAFCO) (Center for Veterinary Medicine, 1995; Quigley, 2011). The AAFCO maintains a list of approved microorganisms for use in animal feed; however, labeling of feed that includes approved probiotic strains only requires the scientific name of the bacterial species rather than specific strain (Quigley, 2011). Probiotics used in feed for livestock in the United States are not approved animal drugs and therefore cannot be marketed with health, structure, or function claims (Center for Veterinary Medicine, 1995). In most cases, probiotic strains approved by AAFCO for use in feed have obtained Generally Recognized as Safe (GRAS) status through scientific review by a panel of experts; however, this practice has received criticism in recent years resulting in a new bill now moving through the United States Congress that would require the Food and Drug Administration (FDA) to review all GRAS submissions (Markey et al., 2022). Although the AAFCO maintains an approved probiotic strain list, oversight and testing of direct fed microbials in the United States varies among regulatory agencies. Consumers might consider verifying both probiotic species and concentration (live colony forming units) contained within purchased probiotic products. It should be noted that the AAFCO Official Publication is not a free publicly available document; membership in AAFCO or a fee must be paid to gain access.

In the United States, regulatory oversight for probiotic interventions has been historically inconsistent with multiple regulatory bodies in charge of maintaining and ensuring compliance efforts. The United States generally follows an assumption of safety principle regarding new products in the market. Controlled clinical data investigating the effects of probiotics are often not available to help guide regulatory policy (Metlay et al., 2006), and the variability in application strategies across livestock, plant, and human subjects complicates regulatory approval processes. The FDA, Environmental Protection Agency, and U.S. Department of Agriculture recently launched a Unified Website for Biotechnology Regulation to streamline information about the three regulatory agencies charged with overseeing agriculture biotechnology products (Commissioner O of the. FDA, 2020). The website describes the federal review process for certain biotechnology products and provides enhanced customer service to innovators and developers to improve transparency, predictability, coordination, and efficiency of the biotechnology regulatory system (,). Additionally, in 2017, the United States implemented the veterinary feed directive (VFD), designed to regulate the use of antibiotics deemed medically important to human medicine (Ferry, 2020). Preliminary data show a 38% decline in domestic antibiotic sales according to a 2018 report by the FDA (FDA C for VM, 2019), suggesting fewer antibiotics are being used in the United States livestock sector.

## Canada

Viable microbial products (VMPs) such as probiotics may be supplied to livestock as livestock feed (similar to direct-fed microbials in the United States), veterinary drugs, or veterinary biologics (Canadian Food Inspection Agency, 2022). The Canadian Food Inspection Agency (CFIA) regulates the manufacture, sale, and import of viable microbial products in Canada including novel livestock feeds that incorporate microorganisms and/or products derived from microorganisms. To be included in livestock feed, microorganisms are required to be listed in CFIA’s Feed Regulations (Schedule IV Part II); however, this document is only available upon written request (Canadian Food Inspection Agency, 2021). Microorganisms that are not included in the Feed Regulations list of approved microorganisms or possessing novel traits must be assessed for safety and efficacy including strain identification, pathogenicity, antimicrobial production, antimicrobial resistance, and specific product specifications that demonstrate quality control (Food Directorate of Health Products and Food Branch, 2022) following the “precautionary principle” requiring proof of safety. Once microorganisms have been deemed approved for use in livestock feed, the safety and efficacy of the intended effect must be supplied by the product manufacturer or owner. However, evidence required for the benefits and use of VMPs in livestock feed is relatively low at one controlled efficacy trial (Canadian Food Inspection Agency, 2022). Increased usage of VMPs in livestock feed is expected in Canada following the December 1, 2018 ruling limiting the availability of medically important antimicrobials for veterinary use to prescription only (Health Canada, 2018).

## European Union

Countries that fall under European Union (EU) jurisdiction follow guidance outlined by the European Food Safety Authority for the regulation and authorization of probiotics for livestock animals. Probiotics which provide a positive effect for the gut microbiome of livestock animals are termed “zootechnical additives” and are regulated as such. Zootechnical additives require a minimum of three studies (short- or long-term) demonstrating efficacy prior to market authorization (European Commission, 2008). Extensive investigation into the specific genome, functional capabilities, and safety of the specific probiotic strain to be included in feed is required for authorization and continued use. This includes identity testing, antimicrobial resistance testing, and virulence testing among others (European Commission, 2008). Additionally, products containing the probiotic must be labelled following established guidance (European Commission, 2008). The EU is well known for applying the “precautionary principle” in regards to new products; however, regulations governing the addition of probiotics to feed are less stringent if the bacterial species of interest has a known safe history of use with a qualified presumption of safety (QPS) (Bernardeau and Vernoux, 2013), a term generated in 2013 to obtain additional safety criteria for all bacterial supplements. To be granted QPS status, a microorganism must have a well-defined taxonomic identity, an established safety record, a substantiated lack of pathogenic properties, and a clearly defined intended use (European Food Safety Authority, 2020). The EU banned in-feed antibiotic use for livestock growth promotion in 2006, and follow up surveillance efforts have identified significant reductions in ARG prevalence in pigs sampled across Europe as a result (Xiao et al., 2016).

## Brazil

As the top beef exporter in the world (Zia et al., 2019), Brazilian regulations may have a large impact on environmental AMR as it relates to livestock agriculture. Products containing probiotics are required to be registered with the health authority (Ministry of Agriculture, Livestock and Food Supply, MAPA, for animal products) (Fonseca and United States Department of Agriculture- Foreign Agricultural Service, 2019). However, detailed information regarding actual use and sourcing requirements of probiotics in agriculture is difficult to access and often behind a paywall. Recently, however, the National Health Surveillance Agency (ANVISA) of Brazil published the “Guide for Procedural Instruction for Probiotic Assessment Request for Food Use,” as a template to help companies register probiotic strains intended for use in food and food products sold in Brazil. The scope of ANVISA’s evaluation includes verification of the identity of the probiotic, and a review of claims regarding the safety and potential benefits of the probiotic strain. It is assumed this guide will soon be used to evaluate probiotics for animal agriculture in the country as well; however, there is no timeline on its implementation. Therefore, more information on the state of probiotics in Brazilian agriculture is necessary for a full assessment.

## China

Animal feed containing probiotics that is sold in China must be registered with the Ministry of Agriculture and Rural Affairs (MARA) as does the product manufacturing facility (United States Department of Agriculture, 2020). Significant trust and reliance are placed on probiotic product manufacturers and manufacturing facilities to ensure strain safety and efficacy; therefore, MARA determines safety at the product level, not the probiotic strain level. Furthermore, probiotic safety statements from regions such as the United States and the EU normally suffice for sale in China. However, as a major importer and exporter of agricultural products worldwide, more clarification on the regulations governing probiotic use in livestock from China would be beneficial.

## The importance of probiotic strain identity and sourcing – a proof of concept in the United States

*Bacillus subtilis* is one of the best studied bacterial species in the field of microbiology, serving as a model for investigating antimicrobial and enzyme production, cell signaling cascades, and the effects of host microbe interactions. For these reasons, the United States Food and Drug Administration (U.S. FDA) has classified this species as safe for human consumption. Though the potential benefits associated with *Bacillus* are well documented (Caulier et al., 2019; Plaza-Diaz et al., 2019), the reproducibility of these interventions largely depends on the *Bacillus* species and strain used (Luo et al., 2019; Wieërs et al., 2020). *Bacillus* strains are remarkably diverse and shaped both by their environment and their ability to acquire genes from closely related species (Earl et al., 2007; Earl et al., 2012). Therefore, due to this species’ diverse ecological range, it is perhaps not surprising that strain specific genetic elements have evolved. Work by Steinke et al. demonstrated that phylogenetically related *B. subtilis* strains share common secondary metabolite biosynthetic gene clusters (Steinke et al., 2021) despite being sampled from different environments. In contrast, work by the Kovácsa Lab discovered that non-ribosomal peptide production varied among *B. subtilis* strains co-isolated from the same soil samples due, in part, to missing core genes and nonsense mutations (Kiesewalter et al., 2020). Furthermore, use of DNA/protein homology search programs such as NCBI BLAST to ‘identify’ secondary metabolite genes/gene clusters and thereby to predict their metabolic products stems from genome annotation issues whereby homology between genes and operons is wrongly interpreted as indicating identical functionality (Klimke et al., 2011). In fact, lipopeptides produced by *Bacillus* can exhibit multiple environmentally driven structural configurations with yet to be determined mechanisms of action (Théatre et al., 2021).

Obtaining basic *in vitro* data related to the identification, gut survival and colonization ability, pathogenicity, toxicity and other safety measures at the strain level, not just species level, is necessary for a potential probiotic (Bernardeau and Vernoux, 2013; Hoffmann et al., 2014; Food and Agriculture Organization of the United Nations, 2016). Bahaddad and colleagues proposed a comprehensive process for identifying and screening *Bacillus* probiotics in monogastric systems (Bahaddad et al., 2022); however, we would like to emphasize the importance of sourcing these strains to ensure both safety at the host level and at the microbial level. Advances in imaging (Son et al., 2015) and analytical (Schindelin et al., 2012) technologies can be used to determine how bacteria adapt and mutate in real time and reveal how the behavior and evolution of probiotics change under antibiotic pressure, when interacting with other gut microflora, and when in direct contact with pathogens (Baym et al., 2016; Zheng et al., 2017). For a thorough review on microbiome transfer opportunities and implications within livestock species we encourage the reader to refer to Brugman et al (Brugman et al., 2018).

Despite efforts to improve our understanding of the *B. subtilis* genome and its plasticity in different environments (Brito et al., 2018; Wu et al., 2021), this knowledge has not been adopted by key governmental bodies regulating probiotic products. According to the U.S. FDA, *B. subtilis* are generally recognized as safe for human consumption. However, as strain level information becomes more accessible, and the risk of antibiotic resistance increases, claiming safety at the species level may not be sufficient (Elshaghabee et al., 2017). One approach would be to evaluate each strain recommended for GRAS certification for the presence and mobility of antibiotic resistance genes prior to commercialization. This analysis and review process could reduce transmission of ARGs thereby protecting public health. It should be noted that specific probiotic species are not required to obtain GRAS status to be included on the AAFCO list for use in animal feed; differences between United States regulations and other regions in this review are detailed in **Table 1**.

## Economic feasibility of probiotics used for disease treatment and prevention in livestock

With increased federal regulations surrounding antibiotic use such as the 2017 United States “Veterinary Feed Directive,” and the 2006 European Union “Ban on Antibiotics Used for Livestock Growth Promotion,” probiotics are experiencing a renaissance. Both farmers and veterinarians are investigating how these therapies can benefit their production systems and practices (Cameron and McAllister, 2019). There is strong evidence to suggest that probiotic supplementation improves the immune response, overall health and performance of livestock, and we encourage the reader to reference Buntyn et al (Buntyn et al., 2016) for a more comprehensive review of these outcomes. Moreover, the value of probiotics is beginning to expand beyond their common mechanisms. Researchers now recognize their ability to mediate viral, fungal, and parasitic infections (Hsu et al., 2018; Saracino et al., 2021; Zuckermann et al., 2022), mitigate the negative effects of heat stress (Jiang et al., 2021), and reduce environmental ammonia and methane emissions generated from livestock and poultry manure (Prenafeta-Boldú et al., 2017; Such et al., 2021). Probiotic strains with these capabilities may then be subsequently applied as fertilizer in adjacent crop fields where they might even improve terrestrial and aquatic ecosystem processes as described earlier.

However, probiotic application is not equally feasible across production systems. Probiotic microorganisms are typically delivered as powders, pastes, or capsules by way of animal feed or drinking water. If water is used, chlorination, temperature, minerals, flow rates, ionophores, and antibiotics must be considered to avoid killing or reducing the effectiveness of the probiotic (Krehbiel et al., 2003). If animal feed is the primary carrier, it is important the microorganisms survive feed processing, especially pelleting, and that the viability of said probiotic is maintained during prolonged feed storage (Krehbiel et al., 2003). Price is another consideration veterinarians and producers debate when determining the appropriate intervention strategy. A 2019 review of Iowa’s swine industry revealed average annual cash expenses associated with a single production site amounted to roughly $730,000 annually (Plastina, 2019). Profit margins for these producers are largely determined by feed conversion ratios, calculated by the relative gain of individual animals with respect to feed intake. An evaluation of probiotic’s ability to successfully and reproducibly improve performance metrics could help farmers understand potential profit margins from probiotic use.

## Conclusions and areas for future research

Bringing a probiotic to market is an extremely complex process involving the evaluation of product safety, efficacy, acceptability, and practicality (Kurt et al., 2019). Most probiotics are marketed to address very specific issues as opposed to the broad-spectrum activity of conventional antibiotics; therefore, it is likely multiple probiotic strains and/or interventions would be needed for the treatment of different infections (Ghosh et al., 2019). Probiotics, despite their potential, will not displace the need for new classes, and sub-classes, of antibiotics (Nwokoro et al., 2016). To effectively utilize probiotics in the feed market and to ensure reproducibility, it is important to understand both the precise mode of action in the gut and to investigate the secondary effects downstream in the surrounding environment. One critical area of research inhibiting a full assessment of the potential for probiotics to reduce environmental AMR is the lack of experimental research investigating the levels of antibiotic residues and resistant bacteria in the environment following a switch to probiotic therapy. Researched aimed at filling this data gap could help determine the effectiveness of probiotics as an antibiotic alternative therapy aimed at reducing environmental AMR.

As the global population increases, the animal agriculture sector will need to consider new sustainable practices to keep pace in feeding the world. One possible option would be to perform more rigorous safety assessments on probiotics intended for livestock similar to those required for human probiotic supplements. Additionally, developing global standards for probiotic screening may be beneficial given the increasing frequency and scale with which animals and animal by-products are internationally bought and sold (Chatellier, 2021). Strain and product level safety information is already a requirement of many countries across the globe prior to probiotic commercialization. As ‘big data’ and machine learning algorithms become more commonplace, developing models intended to predict probiotic outcomes may also be considered in addition to laboratory screening methods to identify unique probiotic features that enhance real world efficacy and reproducibility (Westfall et al., 2021; McCoubrey et al., 2022). This information can then be used to search for and screen new probiotic strains.

Lastly, broader screening for probiotics may find the positive effects are not confined to a small number of bacterial species and genera. Innovative probiotic screening measures without selection bias are under development such as recent work by Li et al. who investigated the dynamic shifts of the swine gut microbiota in an effort to identify novel probiotic microorganisms (Li et al., 2020). Novel probiotics are required to pass safety and efficacy assessments by the FDA prior to commercial production, but evaluation of the ability of the probiotic strain to exacerbate, disseminate, or contribute to the evolution of AMR may be an additional consideration. Nevertheless, as sequencing technologies improve and the cost associated with whole genome and microbiome related studies is reduced, investigations into the long-term effects of currently approved probiotic therapies on the animal microbiota and microbiome are possible and can be considered, particularly in livestock species where performance improvement and disease eradication tactics are urgently needed.

## Author contributions

The authors confirm contribution to the paper as follows: study conception and design: KL and KH. Data collection: KL and RB. Analysis and interpretation of results: KL and RB. Draft manuscript preparation: KL, RB, and KH. All authors contributed to the article and approved the submitted version.

## Funding

75% effort undertaken is sponsored by the Department of the Navy, Office of Naval Research under ONR award number W9132T2220001. For KRL and KRH any opinions, findings, and conclusions or recommendations expressed in this material are theirs and do not necessarily reflect the views of the Office of Naval Research. Any use of trade, firm, or product names is for descriptive purposes only and does not imply endorsement by the U.S. Government.

## Conflict of interest

The authors declare that the research was conducted in the absence of any commercial or financial relationships that could be construed as a potential conflict of interest.

## Publisher’s note

All claims expressed in this article are solely those of the authors and do not necessarily represent those of their affiliated organizations, or those of the publisher, the editors and the reviewers. Any product that may be evaluated in this article, or claim that may be made by its manufacturer, is not guaranteed or endorsed by the publisher.

## Author disclaimer

This work relates to Department of Navy award W9132T2220001 issued by the Office of Naval Research. The United States Government has a royalty-free license throughout the world in all copyrightable material contained herein. Any opinions, findings, and conclusions or recommendations expressed in this material are those of the author(s) and do not necessarily reflect the views of the Office of Naval Research.
